# Synthesis, Nanomechanical Characterization and Biocompatibility of a Chitosan-Graft-Poly(ε-caprolactone) Copolymer for Soft Tissue Regeneration

**DOI:** 10.3390/ma12010150

**Published:** 2019-01-04

**Authors:** Costas A. Charitidis, Dimitrios A. Dragatogiannis, Eleni Milioni, Maria Kaliva, Maria Vamvakaki, Maria Chatzinikolaidou

**Affiliations:** 1 Research Unit of Advanced, Composite, Nano-materials and Nanotechnology, School of Chemical Engineering, National Technical University of Athens, 9 Heroon Polytechniou St., Zographos, 15780 Athens, Greece; ddragato@chemeng.ntua.gr (D.A.D.); milionielen@gmail.com (E.M.); 2 Department of Materials Science and Technology, University of Crete, 71003 Heraklion, Greece; kalivm@iesl.forth.gr (M.K.); vamvakak@materials.uoc.gr (M.V.); mchatzin@materials.uoc.gr (M.C.); 3 Institute of Electronic Structure and Laser, Foundation for Research and Technology-Hellas, 71110 Heraklion, Greece

**Keywords:** nanomechanical properties, chitosan, poly(ε-caprolactone), degradation, soft tissue engineering, Wharton’s jelly mesenchymal stem cells (WJ-MSCs)

## Abstract

Tissue regeneration necessitates the development of appropriate scaffolds that facilitate cell growth and tissue development by providing a suitable substrate for cell attachment, proliferation, and differentiation. The optimized scaffolds should be biocompatible, biodegradable, and exhibit proper mechanical behavior. In the present study, the nanomechanical behavior of a chitosan-*graft*-poly(ε-caprolactone) copolymer, in hydrated and dry state, was investigated and compared to those of the individual homopolymers, chitosan (CS) and poly(ε-caprolactone) (PCL). Hardness and elastic modulus values were calculated, and the time-dependent behavior of the samples was studied. Submersion of PCL and the graft copolymer in α-MEM suggested the deterioration of the measured mechanical properties as a result of the samples’ degradation. However, even after three days of degradation, the graft copolymer presented sufficient mechanical strength and elastic properties, which resemble those reported for soft tissues. The in vitro biological evaluation of the material clearly demonstrated that the CS-*g*-PCL copolymer supports the growth of Wharton’s jelly mesenchymal stem cells and tissue formation with a simultaneous material degradation. Both the mechanical and biological data render the CS-*g*-PCL copolymer appropriate as a scaffold in a cell-laden construct for soft tissue engineering.

## 1. Introduction

Heart failure and myocardial infarction are the most frequent death causes in the world [1]. Surgical interventions result in the treatment and/or limitation of extension of the myocardial infarction. However, the widely used surgical techniques are unable to restore the cardiac function of the heart muscles that have been subjected to death [2]. Also, the limited number of organ donors has led the research interest in investigating alternative methods for heart repair, one of which is the in vitro engineering of myocardial tissue [3,4,5,6]. Tissue engineering requires the use of suitable scaffolds that serve as structural and biochemical templates for cell growth and tissue development [7]. Moreover, scaffolds designed for myocardium tissue engineering should present optimized mechanical, electrical, and morphological properties [8], combined with an appropriate three-dimensional (3D) architecture, in order to engineer the contractile and functional equivalent of the native myocardium [9]. In the literature, various natural as well as synthetic polymeric materials have been studied in terms of their suitability to develop scaffolds for tissue engineering. Chitosan (CS) and poly(ε-caprolactone) (PCL) are among the most widely studied polymers for scaffold fabrication. CS is a natural, highly basic polysaccharide containing reactive amino and hydroxyl groups, which account for its chemical and biological properties [10]. CS is biocompatible, biodegradable to normal body constituents, non-toxic, and cost-effective [11], and has thus been used in a variety of biomedical applications, including drug delivery systems [12,13], absorbable sutures, wound-dressing materials [14], artificial skin, as well as in cardiac tissue engineering [15,16,17], because of its facile processing into porous scaffolds [18]. On the other hand, PCL is a synthetic, semi-crystalline, aliphatic polyester formed by the ring opening polymerization of ε-caprolactone, and presents high biocompatibility and tensile strength and has therefore been used in medical devices [19], in scaffolds for tissue engineering [20] and cardiac tissue engineering, and in drug delivery systems [21]. PCL has a glass transition temperature of about −60 °C, a melting point of ~60 °C, and low viscosity, factors that contribute to its facile processability [22,23].

The last several years there has been a tremendous attempt to blend CS and PCL, although these materials are considered immiscible [24,25,26,27,28]. It is believed that mixing hydrophilic CS with the hydrophobic PCL will allow for the improvement of water diffusion to the proximity of the hydrophobic chains and, thus, accelerate the hydrolytic degradation of the latter [29]. By employing different processing routes, researchers have mixed CS with PCL and have formed 3D structures suitable for cells adhesion, differentiation, and tissue regeneration via an electrospinning process [29,30,31,32,33]. Studies have mainly focused on bone, cartilage, and skin tissue regeneration [29,30,31,32,33], whereas, lately, promising results for cardiac tissue engineering have been also obtained by designing a scaffold with a suitable pore size and mechanical properties, resembling those of the native tissue [22]. However, despite the great interest that these two materials have attracted, there are still many parameters that should be considered, related to the mechanical integrity, the degradation rate, and the effective cell attachment and tissue development on the scaffolds, before their in vivo application.

In the present study, the nanomechanical behavior of a chitosan-*graft*-poly(ε-caprolactone) (CS-*g*-PCL) copolymer was investigated, and its potential use in soft tissue regeneration was assessed. One of our aims was to elucidate the effect of submersion in the cell culture medium, alpha Modified Eagle’s Medium, on the mechanical and physicochemical properties of the copolymer. Moreover, we examined in vitro the viability and proliferation of mesenchymal stem cells (MSCs) derived from the Wharton’s jelly (WJ) of umbilical cords on CS-*g*-PCL copolymer discs up to seven days in culture, and the morphology of the cell-material construct after one and four weeks in culture. To the best of our knowledge, this is the first report on the mechanical properties of a CS-*g*-PCL graft copolymer investigated by nanoindentation in the hydrated and dry states. Ongoing work, on the differentiation of WJ-MSCs under induction with a cardiogenic medium comprising oxytocin, explores the potential of combining the differentiated cells with 3D scaffolds made from this material for use in myocardium tissue engineering applications.

## 2. Materials and Methods

### 2.1. Materials and Synthesis

**Figure 1:**
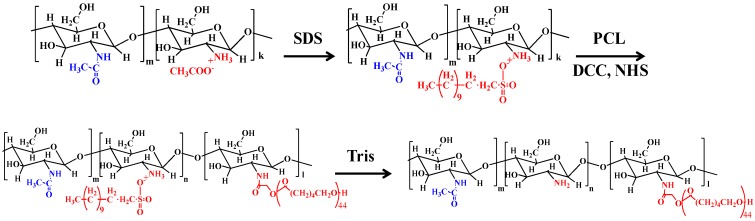
A schematic presentation of the synthetic procedure that was followed for the preparation of the chitosan-*graft*-poly(ε-caprolactone) (CS-*g*-PCL) copolymer.

ε-Caprolactone (ε-CL), CS (molecular weight (*M_w_*) = 110–150 kDa), Tin (II) 2-ethylhexanoate (Sn(Oct)_2_), glycolic acid, sodium dodecyl sulfate (SDS), N,N’ dicyclohexylcarbodimide (DCC), and N-hydroxysuccinimide (NHS) were obtained from Sigma (Steinheim, Germany). All other chemicals and solvents were of analytical grade and were used without further purification. PCL functionalized with one terminal carboxylic acid moiety (PCL-COOH) was synthesized by ring opening polymerization as described previously [34]. In a typical synthesis, ε-CL (10.0 g, 87.6 mmol), glycolic acid (0.22 g, 2.9 mmol), and Sn(Oct)_2_ (0.0048 g, 0.012 mmol) were added into a round bottom flask. The flask was heated at 140 °C for 18 h under a N_2_ atmosphere and continuous stirring. Next, the flask was cooled to room temperature and the obtained solid was dissolved in tetrahydrofuran (THF) and then poured into methanol to remove any unreacted monomer and the catalyst (Sn(Oct)_2_). Finally, the precipitate was collected via centrifugation, washed with methanol, and dried under vacuum to obtain the product, PCL-COOH, in the form of a dry powder. In the second step, the PCL-COOH prepared above was used for the synthesis of the graft copolymer, CS-*g*-PCL (see Figure 1) [30] (Cai et al., 2009). Briefly, the process involved first the formation of an organosoluble complex of CS with SDS (SDS/CS/Complex) (SCC), by simply mixing an acidic solution of CS (1.0 g, 6.1 mmol of the repeating units) in 200 mL of 2% v/v acetic acid) with a solution of SDS (2.0 g, 6.9 mmol) in water under continuous stirring. The mixture was stirred overnight at room temperature, before collecting the precipitate by centrifugation and washing it several times with nanopure H_2_O. The final product was obtained by freeze-drying. Next, the activated ester form of PCL-COOH was prepared by dissolving PCL-COOH (0.2 g, 3.9 × 10^−5^ mol), NHS (9.0 mg, 7.8 × 10^−5^ mol), and DCC (16 mg, 7.8 × 10^−5^ mol) in 50 mL of dimethylformamide (DMF). The reaction mixture was stirred at room temperature under a N_2_ atmosphere for 24 h and then filtered. The filtrate was dried under vacuum to yield the active ester derivative (PCL-COO-NHS), which was then reacted with the amine groups of SCC. In a typical process, PCL-COO-NHS was dissolved in 5 mL N-methylpyrrolidinone and subsequently added into the solution of SCC in dimethyl sulfoxifde (DMSO) under continuous stirring. The reaction was allowed to proceed at room temperature for 48 h under an inert atmosphere and was subsequently dialyzed against DMSO (membrane with molecular weight cut-off MWCO 14000Da) to remove any unreacted PCL-COOH and the excess of NHS and DCC. Next, SDS was removed from the SCC-*g*-PCL graft copolymer by precipitation into a 15% tris(hydroxymethyl)aminomethane (Tris) aqueous buffer solution. The precipitate was washed several times with the 15% Tris solution, DMF, and nanopure water, and was finally lyophilized to obtain the CS-*g*-PCL copolymer in a spongy-like form.

### 2.2. Disc Fabrication

CS, PCL-COOH, and CS-*g*-PCL were pressed into discs with a diameter of 13 mm and a thickness of 1.5 mm using a SPECAC mechanical compacting press (manually operated hydraulic press, SPECAC-LTD, Orpington, UK). A pressure of 15 tons was applied for 3 min for the preparation of the samples.

### 2.3. Characterization Techniques

Attenuated total reflectance-Fourier transform infrared (ATR-FTIR) spectra were recorded on a Nicolet 6700 optical spectrometer (ThermoFisher Scientific, Waltham, MA, USA). For each spectrum, 128 scans were collected in the range of 400–4000 cm^−1^. Proton nuclear magnetic resonance (^1^H NMR) spectra were measured on a Bruker AMX-500 spectrometer (Rheinstetten, Germany). Trimethylsilyl propanoic acid (TSP) was used as an internal standard when the solvent mixture CF3COOD:D_2_O was used. The molecular weight (*M_w_*) and molecular weight distribution (MWD) of poly(ε-caprolactone) were determined by gel permeation chromatography (GPC) (Waters,Waters, Milford, MA, USA). The instrument was equipped with two PL mixed-D and mixed-E columns and a Waters 415refractive index detector operating at 35 °C. Calibration was based on a series of six narrow *M_w_* linear poly (methyl methacrylate) standards ranging from 850 to 342,900 g mol^−1^ and THF was used as the eluent at a flow rate 1 mL min^−1^. The thermal stability of CS-*g*-PCL, PCL-COOH, and CS was investigated by thermogravimetric analysis (TGA) using a Perkin Elmer Pyris Diamond thermogravimetry/differential thermal analyzer (TG/DTA) Perkin Elmer, Llantrisant, UK) instrument. In a typical measurement, ~10 mg of the disc samples were placed in a platinum holder and were heated under constant nitrogen flow from room temperature up to 650 °C at a heating rate of 10 °C/min.

The thermal transitions of CS, PCL-COOH, and CS-*g*-PCL were investigated using a PL-DSC differential scanning calorimeter (PL-DSC, polymer laboratories, Church Stretton, UK). All measurements were carried out under a nitrogen flow, while controlled cooling was achieved using liquid nitrogen. The temperature was varied between −100 °C and 200 °C for CS and CS-*g*-PCL, and between −100 °C and 125 °C for PCL-COOH. In all of the measurements, one cooling and two heating cycles were performed. The first heating cycle was performed to erase the thermal history of the samples, whereas the melting temperature and the enthalpy of fusion (ΔHf) were determined from the second heating cycle for all samples. Furthermore, X-ray diffraction (XRD) measurements were employed to examine the crystallinity of PCL-COOH, CS, and CS-*g*-PCL. XRD patterns were obtained on a PANanalytical X´pert Pro MPD powder diffractometer (Lelyweg, the Netherlands) (40 kV, 45 mA) using CuKa radiation (λ = 1.5418°).

### 2.4. Discoid Sample Degradation

The PCL-COOH and CS-*g*-PCL disc samples were disinfected with 70% ethanol in water, and then weighed for the degradation study. Each sample was incubated separately in a sealed tube with 20 mL α-MEM cell culture medium (pH 7.4) at 37 °C for 3 weeks. The CS-*g*-PCL and PCL samples were removed from the culture medium every 7 days, rinsed with distilled water, and dried for 1 h in an oven at 110 °C and at 37 °C, respectively, to effectively remove the water. Next, the samples were weighted with an accuracy of 0.01 mg before being placed back into a sterile tube with fresh culture medium. The % total weight loss for each sample was calculated after three weeks incubation in the culture medium. The values represent averages of triplicate experiments ± standard deviation (STDV).

### 2.5. Scanning Electron Microscope (SEM)

CS-*g*-PCL and PCL-COOH disc samples, before and after degradation, were sputter-coated with a 10-nm thick layer of gold (Baltec SCD 050, BAL-TEC AG, Balzers, Liechtenstein) and were visualized under a field-emission scanning electron microscope (FESEM, JEOL 7000, Tokyo, Japan) at an accelerating voltage of 15 kV. The samples subjected to a three-week degradation process were first dehydrated in increasing ethanol concentrations and dried in a critical point drier (Baltec CPD 030, BAL-TEC AG, Balzers, Liechtenstein) before being observed by FESEM.

### 2.6. Nanoindentation Testing

Indentation testing of biological tissues and biomaterials possesses many challenges. The nanoindentation theory, analysis, and instrumentation have been developed and validated for smooth, solid, elastic, and elastic-plastic materials; however, biological materials, and in particularly soft tissues, represent a class of porous, hydrated, and viscoelastic materials with irregular geometries [35,36,37,38]. Furthermore, the plastic deformation imposed underneath the indenter, with pile-up or sink-in deformation, results in the misinterpretation of the calculated contact area, and, consequently, the hardness and elastic modulus values [39] are overestimated or underestimated.

The nanomechanical characterization was performed with a Hysitron TriboLab® test instrument (Minneapolis, MN, USA) equipped with a two-dimensional force displacement transducer. The transducer can apply loads in the range of 1–30,000 μN with a resolution of 1 nN, while the maximum penetration depth recorded is 3000 nm (3 μm) with a resolution of 0.04 nm. A Berkovich diamond indenter (100 nm tip radius) was used for the investigation of mechanical behavior of the as-prepared (a) CS, and (b) CS-*g*-PCL disc samples, whereas a fluid conospherical diamond indenter (50 μm tip radius) was used in the case of the α-MEM-submersed disc samples. Experiments were performed in a clean area environment with ~45% humidity and 23 °C ambient temperature. Using the Oliver and Pharr method, hardness (H), elastic modulus (E), and reduced modulus (Er) values were determined for the as-prepared (a) CS, and (b) CS-*g*-PCL samples. Tests at various maximum loads from 50 to 1000 μN with different hold times (5, 10, and 20 s) at the maximum applied load and with an identical 10 s loading and unloading time were performed in order to investigate the time-dependent behavior of the materials. CS-*g*-PCL samples were also immersed in α-MEM at 25 °C and their mechanical behavior was studied after certain time periods (1, 2, and 3 days). However, after immersion in α-MEM, the CS-*g*-PCL sample exhibited lower resistance and stronger adhesion of the tip, and thus the nanoindentation tests were conducted following the displacement control mode at different displacement depths (from 100 nm to 1000 nm), with a 10 s hold time and an identical 10 s loading and unloading time. All H, E, and Er values presented and calculated in this study are an average (±STDV) of six tests conducted in two different disc samples for CS and CS-*g*-PCL. It was also noticed that the CS sample became swollen within the first 2 h of immersion in α-MEM and completely dissolved in the aqueous medium after 24 h, prohibiting measurement of its mechanical properties.

### 2.7. Cell Culture

Wharton’s jelly mesenchymal stem cells (WJ-MSCs) isolation, expansion, and immunophenotypical analysis were performed as described previously [34]. Briefly, umbilical cord was collected after the written informed consent of the donors. Wharton’s jelly was manually scrapped off from the inner lining of the cord, chopped into small pieces, and plated in six-well plates. Adherent WJ-MSCs outgrew from the tissue within 1–2 weeks. Cells were then expanded in α-MEM cell culture medium supplemented with 10% FBS, 2 mM L-glutamine, and 100 IU/mL penicillin/streptomycin (complete α-MEM) (all from Invitrogen, Carlsbad, CA, USA). Subsequently, cells were harvested using 0.25 % trypsin-1 mM EDTA and cultured at a plating density of 1000 cells/cm^2^ for two passages.

### 2.8. Cell Viability and Proliferation

CS-g-PCL and PCL-COOH disc samples were disinfected with ethanol, rinsed briefly with culture medium, and placed in a 24-well plate. Cells (2 × 10^4^) in complete α-MEM were seeded on the samples and placed in the cell culture incubator at 37 °C. Viable cell proliferation was performed with the colorimetric resazurin-based PrestoBlue^®^ assay (Invitrogen, Carlsbad, CA, USA) using a calibration curve [40]. After 1, 3, and 7 days in culture, we removed the culture medium, incubated the samples with the PrestoBlue^®^ reagent at 37 °C for 60 min, and measured the absorbance at 570 and 600 nm in a spectrophotometer (Molecular Devices SpectraMax M2, Molecular Devices, San Jose, CA, USA). We express the cell viability and proliferation as cell number by means of a calibration curve. Error bars represent the average of triplicates ± STDV.

### 2.9. Cell Morphology on CS-g-PCL

A cell suspension of 2 × 10^4^ WJ-MSCs in complete α-MEM was seeded on the CS-*g*-PCL discs and placed in the cell culture incubator at 37 °C for 1 and 4 weeks. The samples were then removed from the incubator and rinsed three times with PBS, fixed with 2% para-formaldehyde for one hour, post-fixed with 1% osmium tetroxide, and dehydrated in increasing ethanol concentrations. The samples were next dried in a critical point dryer (Baltec CPD 030, BAL-TEC AG, Balzers, Liechtenstein), sputter-coated with a 10-nm thick layer of gold (Baltec SCD 050, BAL-TEC AG, Balzers, Liechtenstein), and observed under a scanning electron microscope (JEOL JSM-6390 LV, Tokyo, Japan) at an accelerating voltage of 15 kV.

### 2.10. Extracellular Collagen Production

The levels of total collagen secreted into the culture medium were assessed by a modified Sirius Red Dye assay [41]. Sirius Red is an anionic dye with sulphonic acid side groups reacting with the residual groups of the basic amino acids of collagen. The dye reagent binds specifically to the intact triple helix of mammalian collagen. A volume of 100 μL cell culture supernatant was stained with 1 mL of a 0.1% Sirius Red Dye (Sigma-Aldrich, St. Louis, MO, USA) in acetic acid solution for 30 min. The dye-collagen complex was then precipitated by centrifugation, washed with 0.1 N HCl, and dissolved in 100 μL 0.5 N NaOH. The absorbance was measured by means of a Synergy HTX plate reader at 530 nm. Collagen concentrations were calculated using a standard curve of stained type I collagen with Sirius Red Dye. Samples were measured in triplicates.

## 3. Results and Discussion

### 3.1. Synthesis and Physicochemical Characteristics of CS-g-PCL

**Figure 2:**
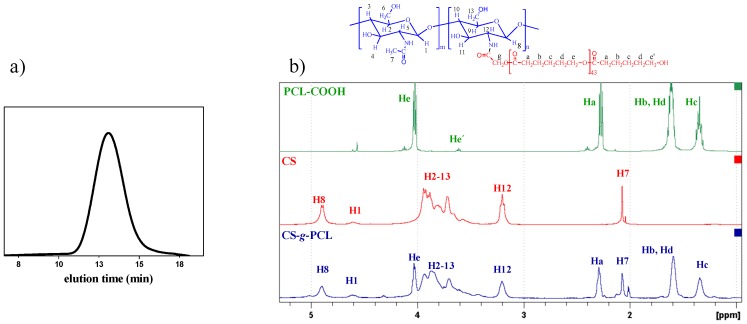
Gel permeation chromatography (GPC) of PCL-COOH (**a**) and ^1^H NMR of PCL-COOH, CS, and Cs-*g*-PCL (**b**).

First, the molecular characteristics of the synthesized PCL-COOH were determined by GPC and ^1^H NMR spectroscopy (Figure 2). The apparent number average molecular weight (*M*_n_) and the polydispersity (*M*_w_/*M*_n_) of PCL-COOH by GPC were found to be 7700 g/mol and 1.78, respectively. A ^1^H NMR analysis allowed for the determination of the absolute molecular weight of the polymer, by end group analysis, which was found to be 5100 g/mol, corresponding to a degree of polymerization (DP) equal to 44 (Figure 2b). The difference between the molecular weights determined by GPC and ^1^H NMR spectroscopy is attributed to the difference of the hydrodynamic volume of PCL compared to that of the PMMA standards used for the GPC calibration.

Next, the successful grafting of the PCL-COOH chains onto the CS backbone was verified by both ATR-FTIR and ^1^H NMR spectroscopies. The appearance of the characteristic bands of the amide groups of CS at 1650 cm^−1^ and 1576 cm^−1^ and the ester group of PCL at 1723 cm^−1^ in the ATR-FTR spectrum of CS-*g*-PCL indicate the successful grafting of PCL-COOH to CS. Furthermore, the appearance of the methylene peaks of PCL at 4.12, 2.40, 1.69, and 1.43 ppm and the broad pyranose hydrogen peaks of chitosan between 4.2 and 3.2 ppm in the ^1^H NMR spectrum of CS-*g*-PCL verifies the presence of both components, PCL and CS, in the synthesized graft copolymer (Figure 2b) [30]. The elimination of SDS from the final product was also confirmed by the disappearance of the sulfate bands at 1200 cm^−1^ and 809 cm^−1^ in the respective ATR-FTR spectrum and of the methylene peaks of SDS in the ^1^H NMR spectrum of CS-*g*-PCL [42,43]. Finally, the degree of grafting of PCL-COOH onto the chitosan polymer chains was calculated from the ^1^H NMR spectrum of the graft copolymer by rationing the peak integrals of the O=C–CH_2_– protons (Ha) of PCL at 2.38 ppm to the pyranose protons (H7) of chitosan at 2.07 ppm, and was found to be 1 chain of PCL per 143 monomer repeat units of chitosan, which corresponds to a 17.2 wt% PCL content of the graft copolymer.

### 3.2. Thermal Properties

**Figure 3:**
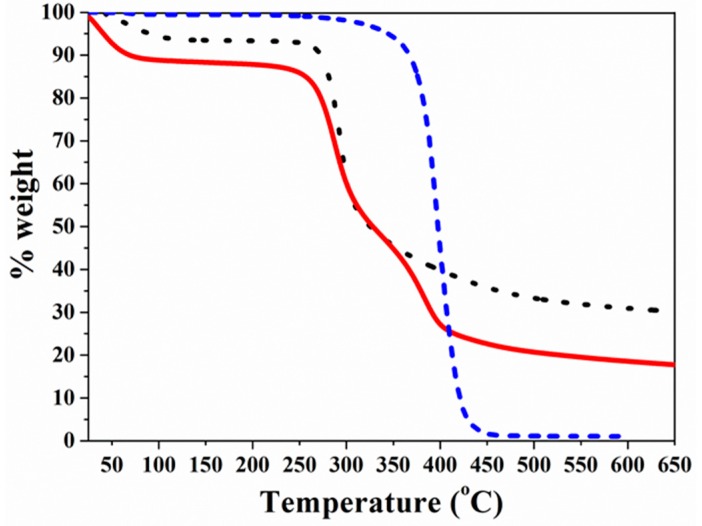
Thermogravimetric analysis curves of discoid samples of CS (⋯), PCL-COOH (– – –), and CS-*g*-PCL (—).

TGA of the disc samples was employed to study the thermal stability of CS, PCL-COOH, and CS-*g*-PCL (Figure 3). The TGA curve of CS shows two sharp degradation processes. The first at 70 °C, which corresponds to a weight loss of 6.6%, is attributed to the absorbed water, while the second at 290 °C, with a weight loss of 52%, is ascribed to the disintegration of CS mainly via a deacetylation and depolymerization process. At temperatures higher than 300 °C, there is a continuous drift in the weight [44,45] up to 600 °C, when 31.4% of the initial material weight remains as residue. On the other hand, the TGA curve of PCL-COOH shows one sharp thermal decomposition process at 400 °C, which is in good agreement with results reported previously in the literature [46]. Finally, upon grafting of PCL-COOH to CS, the TGA curve of CS-*g*-PCL shows three distinguished steps (Figure 3). The first appears at around 43.5 °C and corresponds to 11.1% weight loss, ascribed to the presence of water in the sample. The second step is observed at 285 °C accompanied by a 41% weight loss attributed to the degradation of CS, while the third step at 384 °C corresponds to the decomposition of PCL. At temperatures above 400 °C, the material continues to decompose slowly in a similar manner to that observed for pure CS until 600 °C, when a final residue of 18.8% is obtained. The presence of the thermal decomposition steps of both CS and PCL in the thermal degradation profile of the CS-*g*-PCL copolymer verifies the successful grafting of the PCL-COOH chains onto the CS backbone. Moreover, the TGA curves of CS, PCL-COOH, and CS-*g*-PCL suggest that the PCL in the graft copolymer decomposes at a slightly lower temperature compared to the PCL-COOH homopolymer. This shift was attributed to the grafting of the PCL chains along the CS backbone, which decreases the thermal stability of PCL by possibly reducing its crystallinity as discussed below [47]. Finally, the PCL content of the copolymer was calculated to be 19 wt% by TGA, which is in good agreement with the value found by ^1^H NMR spectroscopy (17.2 wt%).

**Figure 4:**
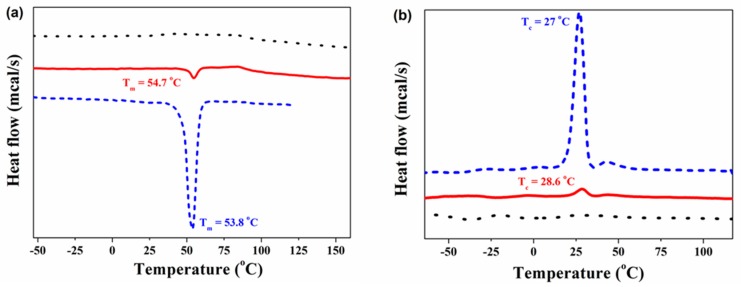
DSC thermograms of CS (⋯), PCL-COOH (– – –), and CS-*g*-PCL (—). The second heating cycle (**a**) and the cooling cycle (**b**).

The thermal transitions of CS, PCL-COOH, and CS-*g*-PCL were also investigated by DSC. Figure 4 illustrates the DSC curves for the CS and PCL-COOH homopolymers, and the CS-*g*-PCL copolymer. The PCL-COOH homopolymer shows a melting temperature (Tm) at 53.8 °C and a crystallization temperature (Tc) at 27 °C, while, the DSC curve of CS does not exhibit any thermal transition in the range −100 to 200 °C. After grafting the PCL-COOH chains onto the CS backbone, a melting and crystallization transition appears for the graft copolymer at Tm = 54.7 °C and Tc = 28.6 °C, respectively. The above transitions are assigned to the PCL chains in the graft copolymer and further verify the successful grafting of PCL-COOH onto the CS backbone. Moreover, the enthalpy of fusion (ΔHf) for the PCL segment in the homopolymer and the graft copolymer was calculated from the respective DSC curves to be 87.86 J/g and 35.75 J/g, respectively. These values correspond to a crystallinity (Xc) of 65.8% for the PCL homopolymer and 26.8% for the PCL in the CS-*g*-PCL copolymer (The ΔHf for 100% crystalline PCL is 133.44 J/g), and suggest a substantial reduction of the PCL crystallinity in the graft copolymer attributed to the complex copolymer architecture [30]. Finally, the lower crystallinity of PCL in the graft copolymer is in good agreement with the TGA results, which showed a lower decomposition temperature for the PCL grafts compared to that found for the PCL-COOH homopolymer.

**Figure 5:**
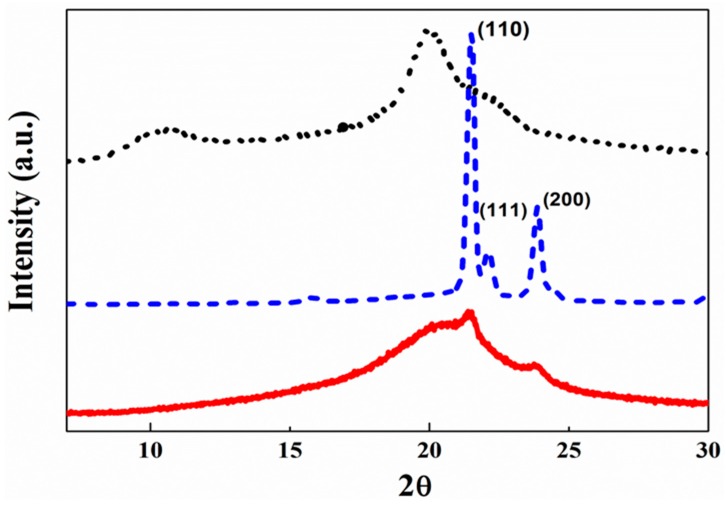
XRD patterns of CS (⋯), PCL-COOH (– – –), and CS-*g*-PCL (—).

XRD measurements were performed to determine the influence of the PCL-grafted chains on the crystallinity of the CS backbone. Figure 5 shows the XRD patterns for PCL-COOH, CS, and the CS-*g*-PCL graft copolymer. The XRD pattern of PCL-COOH shows three strong and sharp peaks at 21.5°, 22.1°, and at 23.9°, which are characteristics for PCL and are attributed to the (110), (111), and (200) crystallographic planes of PCL, respectively [48]. The XRD curve for CS exhibits two characteristic broad peaks at 10.5° and 20.0° corresponding to the crystalline structure of CS and a shoulder at 22.2°, which indicates the presence of amorphous polymer regions [49,50]. Finally, the XRD pattern of CS-*g*-PCL shows the two characteristics peaks of PCL at 21.5° and 22.1°. The intensity of these peaks is significantly reduced compared to those of the PCL homopolymer, verifying the decrease in the crystallinity of PCL in the graft copolymer, in good agreement with the DSC results discussed above. Moreover, the CS peak at 10.5° has disappeared in the XRD pattern of the graft copolymer, while the peak at 20.0° has become much broader, suggesting a decrease of the crystallinity of CS in the graft copolymer due to the presence of the grafted PCL chains along the CS backbone, which hinder the interchain hydrogen bonding interactions and lead to changes in the CS microstructure [9].

### 3.3. Degradation Experiments

Table 1 below shows the weekly weight loss and the total weight loss, after 3 weeks in a-ΜΕΜ, for the PCL-COOH and CS-*g*-PCL samples. Although the two materials exhibit a similar degradation profile for the first two weeks, the total weight loss for the CS-*g*-PCL discoid samples was 35% ± 4%, which is significantly higher compared to that found for PCL-COOH (19% ± 2%) (mean ± STDV, n = 3). The overall higher degradation rate of the copolymer was attributed to the presence of the hydrophilic CS segments, which facilitate water diffusion within the material.

**Figure 6:**
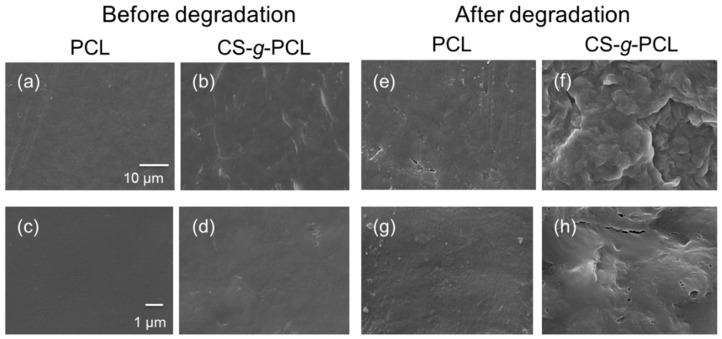
SEM images of the PCL and CS-g-PCL disc samples as prepared (**a**,**c** and **b**,**d**, respectively) and after 21 days of degradation in α-ΜΕΜ (**e**,**g** and **f**,**h**, respectively). Top panel: 2000-fold magnification, scale bar represents 10 μm; bottom panel: 10,000-fold magnification, scale bar represents 1 μm.

SEM images (Figure 6) of the as-prepared PCL and CS-*g*-PCL disc samples (left, Figure 6a–d), and after 21 days in α-MEM (right, Figure 6e–h), were obtained. The as-prepared PCL sample (Figure 6a,c) has a very smooth and uniform surface, whereas the CS-*g*-PCL sample (Figure 6b,d) displays some inhomogeneity and a slightly rough surface attributed to the presence of CS in the copolymer. After 3 weeks of incubation in α-MEM, both the PCL-COOH and CS-*g*-PCL discs undergo significant morphological changes as evidenced by SEM. The PCL material (Figure 6e,g) exhibits an increase in surface roughness and surface inhomogeneity in the form of sparse small holes. On the other hand, the CS-*g*-PLC copolymer sample depicts pronounced morphological alterations and a large increase in surface roughness, whereas small holes and ruptures are observed at a higher magnification (Figure 6f,h). These results are in good agreement with the higher degradation rate of the graft copolymer sample compared to the PCL-COOH homopolymer, discussed above, and was attributed to the more hydrophilic nature of the copolymer and, thus, the higher water uptake.

### 3.4. Nanoindentation Data of as-Prepared Samples

**Figure 7:**
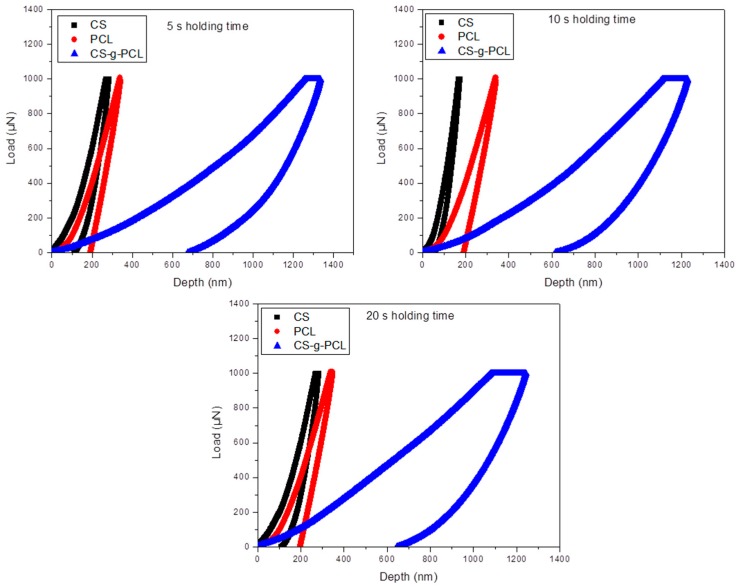
Load-unload curves at 1000 μN maximum applied load with different holding times for the CS, PCL, and CS-*g*-PCL copolymer.

**Figure 8:**
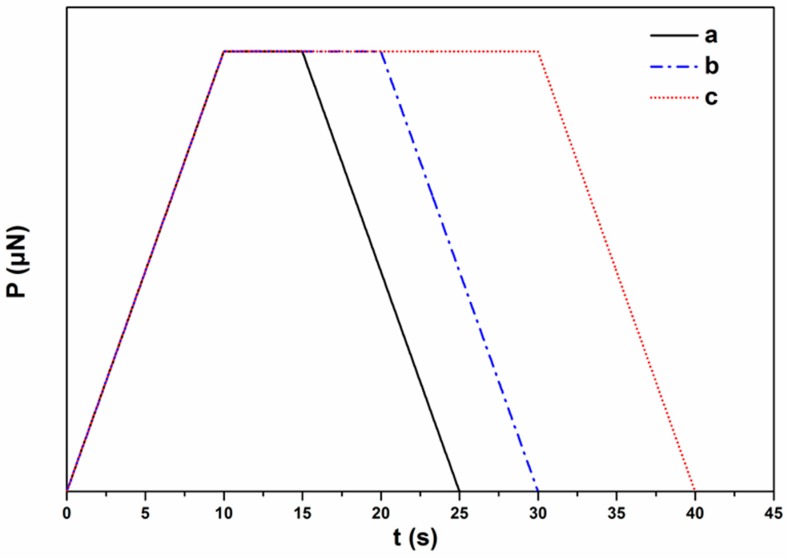
Loading functions for (a) 5, (b) 10, and (c) 20 s holding time at a fixed rise time (10 s).

Nanoindentation tests were performed at various applied loads and holding times reaching different penetration depths as shown in Figure 7. The load control experiments followed a trapezoidal loading-unloading curve depending on the hold time (Figure 8). Figure 7 shows typical load–indentation depth curves for the CS-*g*-PCL and CS samples for three different loading hold times (5, 10, and 20 s) at various maximum depths. Taking into account the geometry of the Berkovich tip, the smooth load-unload curves indicate that there is no significant porosity in the samples synthesized and tested in this study [51]. The load–depth curves for CS-*g*-PCL samples show lower slopes of dP/dh (stiffness values) in the unloading step and higher values of maximum nanoindentation depths compared with pure CS samples. The higher plastic deformation is observed for the CS-*g*-PCL sample, because, at the same applied load, the indenter penetrated the sample at a higher indentation depth followed by higher dissipation of energy compared to the CS sample. The dissipation energy, as a quantitative reflection of the viscous effect, is due to the internal friction or plastic deformation energy inside the copolymer. The lower stiffness for the CS-*g*-PCL sample is attributed to the lower degree of crystallinity of CS chains in the graft copolymer. A similar effect has been reported earlier by [10], which claimed that blending of CS and PCL occurs via hydrogen bond formation between the functional groups present in the CS molecule (–NH_2_ and –OH) and the carbonyl groups of PCL, which also resulted in the suppression of the PCL crystallization [10,24,30,33,52].

**Figure 9:**
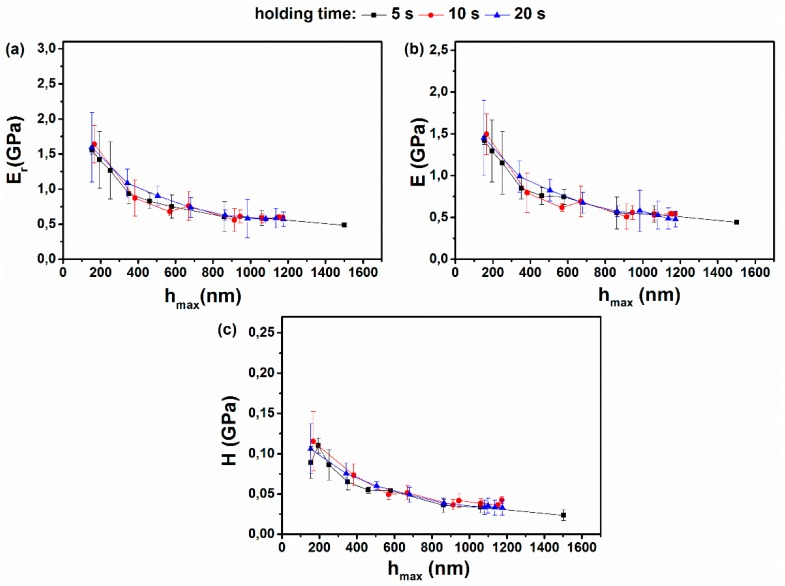
(**a**) Reduced modulus (Er); (**b**) Young’s modulus (E); and (**c**) hardness (H) values as a function of maximum indentation depth for 5, 10, and 20 s holding time for the CS-*g*-PCL copolymer.

Figure 9 presents hardness (H), Young’s modulus (E), and reduced modulus (Er) values for the CS-*g*-PCL copolymer determined at various maximum loads from 5 μΝ to 1000 μΝ, for 5, 10, and 20 s holding time, respectively, following the load control mode. In all three cases, the H, E, and Er values are higher at low penetration depths, whereas as the indenter penetrated further into the sample, the values decreased and finally reached a constant minimum value. It should be noted that the H, E, and Er values for low indentation depths have higher error bars probably due to the surface roughness that affects the calculation of the real contact area. As discussed above in Figure 7, the CS sample presented the higher resistance to applied loads and possessed the highest H and E values compared to the other samples (see Table 2). The high E value for CS and its minimum influence as a function of loading rate was attributed to the formation of interchain hydrogen bonds and the higher crystallinity of the CS homopolymer. On the other hand, the CS-*g*-PCL copolymer exhibited lower E values due to the lower degree of crystallinity of the CS chains as discussed above. Overall, it was found that the graft copolymer presented lower nanomechanical properties compared to the CS and higher compared to PCL-COOH homopolymer samples (Table 2). CS revealed higher H and E values compared to PCL, which was attributed to hydrogen bonding interactions between the amino and the hydroxyl functionalities of the polymer (Table 2). Ιn Table 3, elastic modulus values of biomaterials investigated for myocardial tissue engineering are listed. Some of them are much stiffer, and some others, such as collagen gels, are too weak compare to myocardium tissue. The CS-*g*-PCL sample investigated in this study is a promising material for myocardium tissue engineering, since it is stiffer than myocardium tissue in dry state, degradable (1–2 months), and presents elastic values (≤1 kPa) very close to myocardium tissue in wet state.

### 3.5. Influence of the Creep Time on the Elastic Modulus and Hardness Values

**Figure 10:**
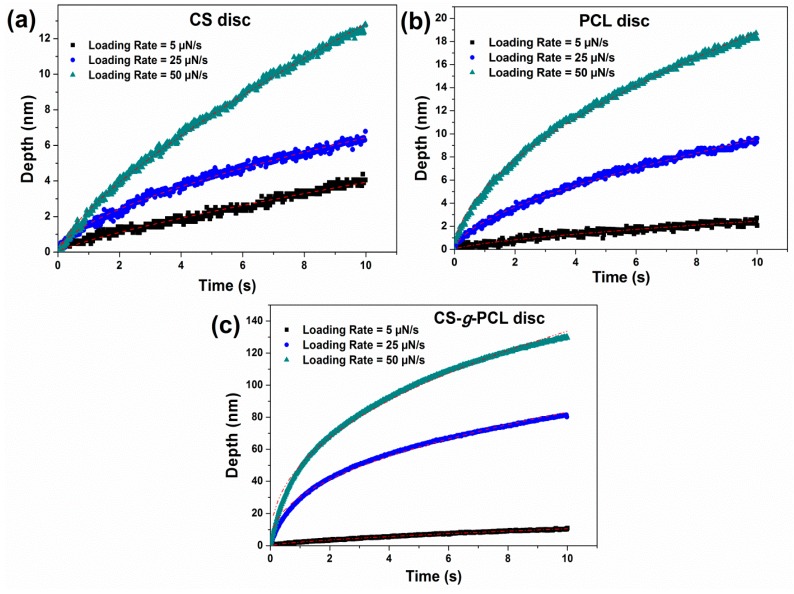
Evolution of penetration depth as obtained at different loading rates and at a constant applied load, as a function of time, for the (**a**) PCL; (**b**) CS; and (**c**) CS-*g*-PCL samples.

The tests were performed under three different holding periods (creep time) (5, 10, and 20 s) at maximum applied loads to study the creep behavior and the effect of holding time on the unloading segment of the CS-*g*-PCL and CS samples (Figure 10). Figure 9 illustrates that the creep holding time has a minor influence on the H, E, and Er values calculated from the load-unload curves. Furthermore, the increase in the penetration depth during the load holding step is due to the viscous effect [57] (Oyen and Cook, 2003). PCL has a glass transition temperature (Tg) at −60 °C, which is lower than that of CS (140–150 °C) [23,58] (Sinha et al., 2004; Dong et al., 2004). Since the nanoindentation tests were performed at room temperature, which is above the Tg of PCL, the chains are free to relax when a constant stress is applied, whereas the CS sample is below its Tg value and the CS chains cannot relax and present any creep deformation. The higher indentation displacement during creep was observed for the CS-*g*-PCL copolymer, which was attributed to the low Tg of the PCL side chains and their lower degree of crystallinity in the graft copolymer as discussed above [10,24,30,33,52]. For amorphous polymers or amorphous regions, the free volume of a molecular chain is so small at a temperature below Tg that the creep due to molecular motion should not be critical [59].

### 3.6. Nanoindentation Analysis of the Samples Following Immersion in a-MEM

**Figure 11:**
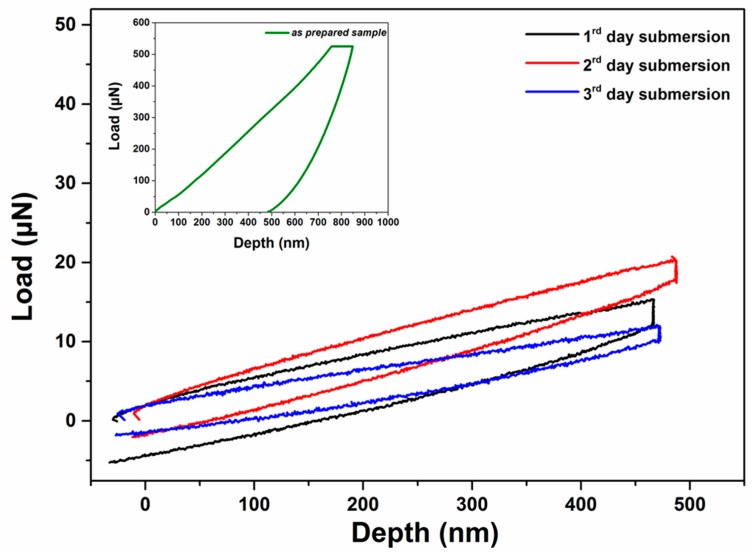
Load-unload curves for the CS-*g*-PCL sample after submersion for 1, 2, and 3 days in α-MEM at 25 °C. The inset shows the load-unload curve for the as-prepared sample.

**Figure 12:**
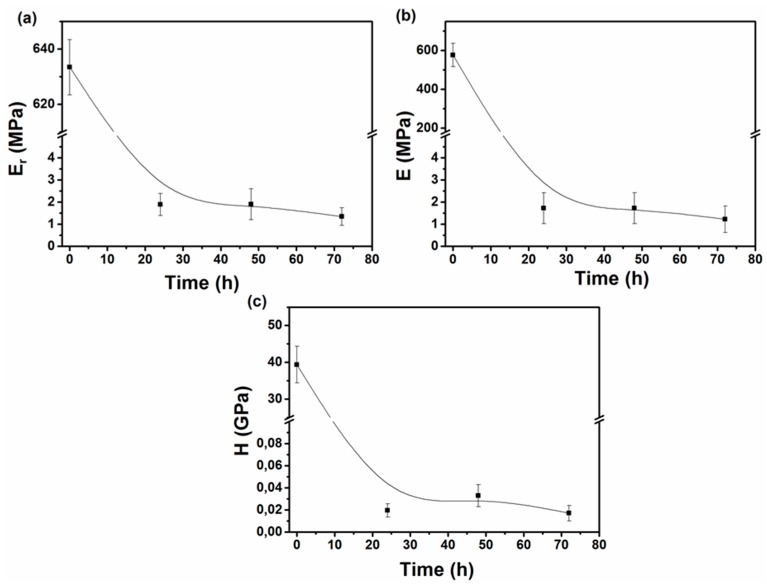
(**a**) Reduced modulus (Er); (**b**) Young’s modulus (E); and (**c**) hardness (H) values for the CS-*g*-PCL sample after submersion in α-MEM.

Following the nanoindentation tests performed on the as-prepared samples, all samples were submersed in α-MEM serum at 25 °C under static conditions, for a period of 3 days, and their nanomechanical properties were evaluated at certain time periods. As already stated above, the hydrophilic nature of CS resulted in the dissolution of the sample within 24 h after submersion in the medium. The CS-*g*-PCL copolymer sample became swollen and fractured after 4 weeks of immersion in the aqueous medium due to the presence of the hydrophilic CS component. Figure 11 shows the load-unload curves for the CS-*g*-PCL copolymer sample after the 1st, 2nd, and 3rd day of submersion in α-MEM. In Figure 12a,b the Young’s modulus and hardness values, calculated using the Oliver and Pharr model for the CS-*g*-PCL samples, respectively, are presented as a function of submersion time. As seen in Figure 12a, the Young’s modulus values for CS-*g*-PCL decrease rapidly in the first few hours of submersion due to the water uptake by the sample. In general, the kinetics of the scission of the ester bonds of aliphatic polyesters controls their hydrolytic rate, and an accelerated degradation is found in amorphous regions where the water molecules can penetrate faster within the disordered network of polymer chains compared to the crystalline ones [60,61]. It would have been expected that the PCL sample would not have revealed such a profound decrease of the measured nanomechanical properties due to its high crystallinity and its hydrophobic nature, which render it resistant to hydrolytic degradation. However, porosity has been stated to regulate the degradation rate of these materials [62,63]. Two different mechanisms have been proposed for the effect of porosity on the degradation rate of PCL. Firstly, it is believed that low porosity results in fast degradation of PCL, PLGA, and PLA because of the internal acid autocatalytic effect [64]. On the other hand, Zhang et al. revealed that increasing the porosity of a PCL scaffold can result in a fast degradation rate, i.e., high weight, molecular weight, and compressive modulus loss, because water inside the bulk structure of the porous PCL scaffold leads to hydrolysis and random chain fracture [65]. For the PCL sample studied herein, it cannot be assumed that porosity results in the decrease of the measured nanomechanical properties, because porosity measurements, by N_2_ adsorption/desorption experiments, on the samples suggested that none of the materials used in this work exhibited any significant porosity. The fast degradation rate of the CS-*g*-PCL disc is attributed to the swelling of the material due to the presence of the hydrophilic CS component and the lower crystallinity of the PCL chains in the graft copolymer. However, despite the softening and the high degradation rate of the CS-*g*-PCL sample, it was observed that the graft copolymer presented adequate mechanical integrity under the applied load at the fully hydrated state for a period of 3 days.

### 3.7. In Vitro Performance

**Figure 13:**
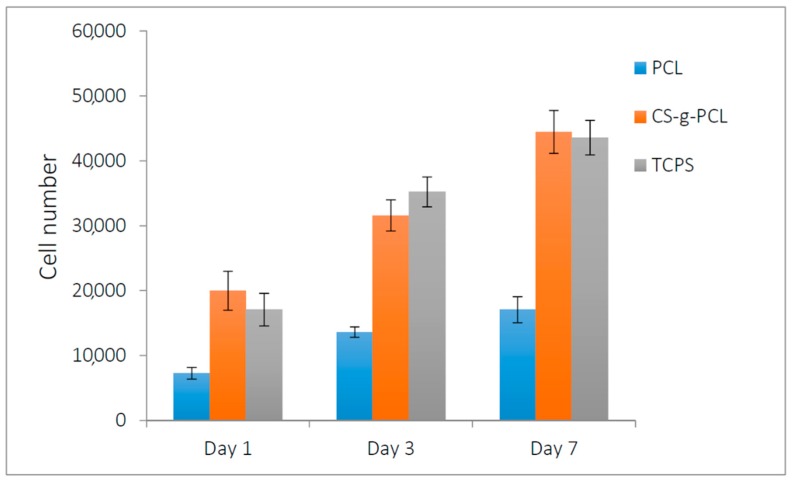
Wharton’s jelly mesenchymal stem cells (WJ-MSCs) viability and proliferation on PCL and CS-*g*-PCL discs and the tissue-culture-treated polystyrene (TCPS) control after 1, 3, and 7 days in culture, by means of the colorimetric assay PrestoBlue® and expressed in cell numbers. Error bars represent the average of triplicates ± standard deviation (STDV).

One of the first biocompatibility-related questions arising for the CS-*g*-PCL discs is whether they support cell adhesion, viability, proliferation, and tissue growth in vitro. We therefore investigated the viability of WJ-MSCs on CS-*g*-PCL discs following the good attachment of the cells on the material, and the cell proliferation after 3 and 7 days of culture. In a previous study, we showed a strong initial adhesion of WJ-MSCs seeded on two-dimensional films of the CS-*g*-PCL copolymer, and depicting a well-spread, flattened cell morphology onto the substrate [34]. Here, we show the viability of WJ-MSCs on PCL and CS-*g*-PCL discs as well as on a tissue-culture-treated polystyrene (TCPS) control after 1, 3, and 7 days in culture (Figure 13). Cells exhibit a proliferation increase after 3 and 7 days on both materials, with a more pronounced increase on the CS-*g*-PCL discs, for which more than a 2-fold increase in proliferation is found. These values are comparable to the TCPS control, and in accordance with previously reported results on CS-*g*-PCL copolymeric films [34]. On the PCL samples, the number of viable cells measured on day 1 is lower compared to the cell number on CS-*g*-PCL due to the hydrophobicity of PCL, which prohibits strong cell adhesion [66]. Similarly, a higher increase in cell proliferation for a CS–PCL blend compared to pure PCL was observed in scaffolds fabricated by melt stretching and multilayer deposition, employing pre-osteoblasts for bone tissue engineering [67]. Chitosan is well-known to favor tissue growth, especially in cardiac tissue regeneration [15,17].

**Figure 14:**
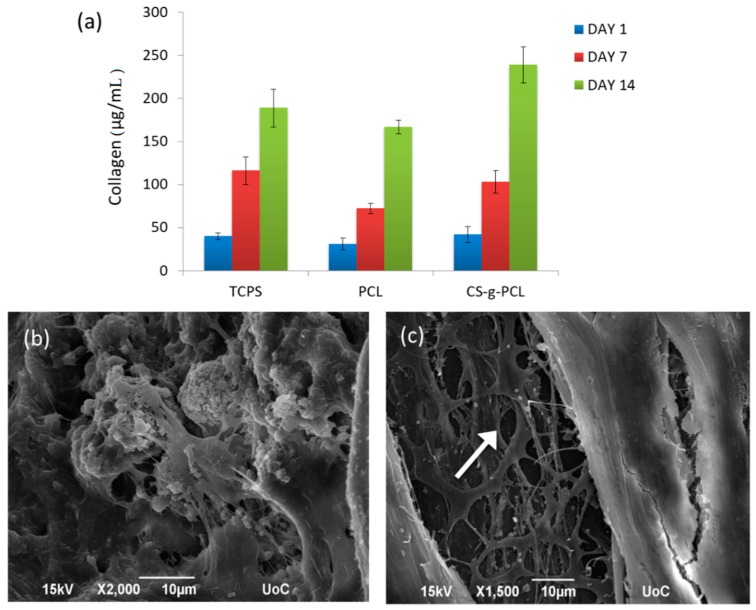
Extracellular collagen production of WJ-MSCs cultured on PCL and CS-*g*-PCL discs and the TCPS control for 1, 7, and 14 days (**a**). SEM images showing WJ-MSCs on CS-*g*-PCL discs after 1 (**b**) and 4 (**c**) weeks in culture. Increased levels of produced collagen are found on all surfaces after 14 days in culture compared to day 1, with the CS-*g*-PCL discs depicting the highest increase. After one week in culture, we observe a high number of cells infiltrated into the pores of the CS-*g*-PCL discs. After four weeks in culture, we observe successive degradation of the CS-*g*-PCL disc (white arrow).

Figure 14 shows the levels of total collagen secreted by the WJ-MSCs cultured on PCL and CS-*g*-PCL discs and the TCPS control for 1, 7, and 14 days (Figure 14a). Representative SEM images depict WJ-MSCs grown onto the CS-*g*-PCL discs after 1 and 4 weeks in culture. We observe a dense network of well-adhered cells with spread morphology (Figure 14b) that are interconnected and cover the surface of the copolymer through a strong attachment onto the material. Interestingly, after four weeks in culture, we observe successive degradation of the CS-*g*-PCL disc (white arrow) (Figure 14c). Following cell infiltration into the pores of the discoid substrate, cells proliferate over a period of two weeks towards a new tissue formation as demonstrated by the production of extracellular collagen. This is evidence of supported cell growth into tissue upon degradation of the material. Chitosan constructs, among different naturally derived hydrogels used for chondrogenesis, have been shown to accumulate the highest levels of sulfated glycosaminoglycan (sGAG), and synthesize the highest levels of collagen, both markers of the extracellular matrix (ECM) formation [68]. Similarly, pre-osteoblastic cells cultured on CS-*g*-PCL films indicated an increased collagen production after 7 days, as well as significantly enhanced matrix biomineralization and osteopontin levels after 14 days compared to the TCPS control [69].

Our results on the proliferation increase of cells cultured on CS-*g*-PCL discs after one week, and the formation of a dense tissue after four weeks accompanied by the successive degradation of the substrate, set the first in vitro study that renders the proposed cell-material construct suitable for tissue engineering applications. Still, for a long-term goal in a cardiac tissue application, there are several biological and functional related issues to be investigated. It is reported that a scaffold for cardiac repair should provide mechanical support and physical and biological cues and be degradable and capable of integration with host tissue [70]. Additionally, a construct should enhance cell viability, proliferation, and functionality and strengthen mechanical and cardiac function. As both the stiffness and the composition of the scaffolds are important in regulating cell behavior and can have complex synergistic effects, ECM-based scaffolds with tunable biochemical and mechanical properties have been reported to be beneficial for cardiovascular tissue engineering [71]. Decellularized human myocardial ECM sheets are able to serve as scaffolds, displaying higher viability than in standard cultures [72]. Also, ECM synthesis and deposition by cardiomyocytes within such scaffolds is important. The prominent role of scaffold composition and architecture in influencing cardiomyocyte phenotype, matrix synthesis, and cytokines release has been reported recently [73]. Polysaccharide-based natural polymers, such as chitosan, have been widely used for heart tissue engineering; however, they still face many challenges, such as the choice of cell type and biomaterial, the vascularization of constructs, the animal models, the timing of treatment, and the route of administration [17].

According to the principle of tissue engineering, the degradation rate of a scaffold should be slower than the targeted tissue formation rate, since the strength of the scaffold must be sufficient to support the regeneration process. In our case, the degradation rate of the copolymer discs with a weight loss of 11% after one week, 18% after two weeks, and 35% after three weeks, could be beneficial for the formation of a soft tissue. Additionally, regenerating tissues require interaction with ECM components not only for mechanical support but also to acquire and maintain appropriate phenotypic and functional characteristics. Therefore, our future work will focus on the differentiation of the WJ-MSCs towards cardiac cells on the copolymer scaffolds, and the investigation of their in vitro and in vivo functionality.

## 4. Conclusions

A CS-*g*-PCL graft copolymer was synthesized and used for scaffold fabrication for soft tissue regeneration. The graft copolymer exhibited a lower degree of crystallinity of the PCL and CS chains compared to that found for the two respective homopolymers. This resulted in lower H and E values and a time-dependent behavior of the mechanical properties due to the presence of PCL, but still sufficient integrity under mechanical stress after 3 days of degradation in the cell culture medium α-MEM at 25 °C. The measured E values are close to these reported for soft tissues, rendering the copolymer a suitable candidate for the growth of WJ-MSCs. The in vitro biological evaluation of the materials clearly demonstrated that the CS-*g*-PCL discs support the growth of WJ-MSCs and lead to a larger than 2-fold increase in proliferation after 3 and 7 days in culture, compared to the PCL samples, and favor tissue formation in culture as indicated by the extracellular collagen production with a simultaneous material degradation. Ongoing investigations on the differentiation of WJ-MSCs to cardiac cells onto 3D CS-*g*-PCL scaffolds using oxytocin for cardiogenic induction could provide a very promising cell-scaffold construct for myocardium tissue engineering. For this long-term objective, the electrical properties of the graft copolymer should be also studied, as well as the ability of the CS-*g*-PCL copolymer to induce synchronized beating with the native cardiac tissue.

## Figures and Tables

**Table 1:** Weight loss and % weight loss for PCL and CS-*g*-PCL in α-MEM.

Days	PCL		CS-*g*-PCL	
	Weight (g)	% Weight Loss (%)	Weight (g)	% Weight Loss (%)
0	0.135 ± 0.003	0	0.082 ± 0.002	0
7	0.120 ± 0.002	11 ± 2	0.073 ± 0.002	11 ± 4
14	0.114 ± 0.002	16 ± 2	0.067 ± 0.002	18 ± 4
21	0.110 ± 0.001	19 ± 2	0.053 ± 0.002	35 ± 4

**Table 2:** Young’s modulus and hardness values of the tested samples at a 1000 nm indentation depth.

Sample	Elastic Modulus (MPa)	Hardness (MPa)
PCL	443 ± 44	13 ± 1.5
CS	615 ± 55	54 ± 5
CS-*g*-PCL	550 ± 88	45 ± 4

**Table 3:** Young’s modulus values of the tested samples and biomaterials used in myocardium engineering.

Sample	Elastic Modulus in Dry State (MPa)	Ref.
CS-g-PCL	550	This Study
PGS	0.04–1.2	[53]
PGA	700–1000	[53]
Collagen gel	0.002–0.022	[54]
PLLA	1200–2700	[55]
Myocardium of Human	0.02–0.5	[56]
